# 

**Published:** 1894-06-16

**Authors:** 


WITH EXTRA NURSING SUPPLEMENT.

Per Annum, 10s. 6d., post free.

Monthly Parts, 6d.; post free, 8d.

Ditto per Annum, 8s. 6d., post free.

### THE HOSPITAL

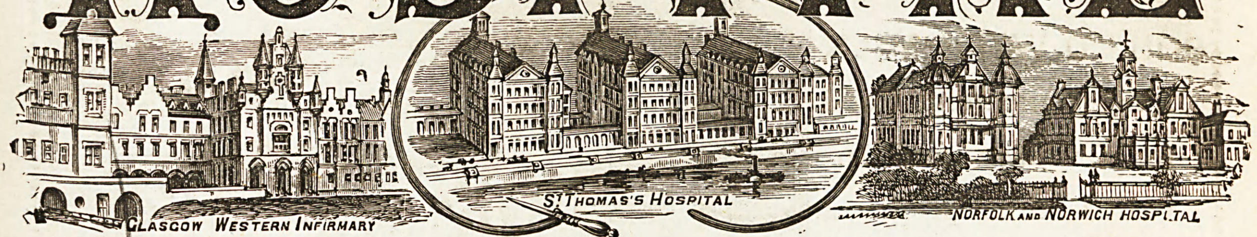

No. 403. Vol. XVI. WITH EXTRA NURSING SUPPLEMENT. SATURDAY, JUNE 16TH, 1894.

